# An Advax-Adjuvanted Inactivated Cell-Culture Derived Japanese Encephalitis Vaccine Induces Broadly Neutralising Anti-Flavivirus Antibodies, Robust Cellular Immunity and Provides Single Dose Protection

**DOI:** 10.3390/vaccines9111235

**Published:** 2021-10-23

**Authors:** Tomoyoshi Komiya, Yoshikazu Honda-Okubo, Jeremy Baldwin, Nikolai Petrovsky

**Affiliations:** 1 Faculty of Health and Medical Sciences, Hokuriku University, Kanazawa 920-1180, Japan; t-komiya@hokuriku-u.ac.jp; 2 Vaxine Pty Ltd., Adelaide 5046, Australia; yoshikazu.hondaokubo@flinders.edu.au (Y.H.-O.); jeremy.baldwin@flinders.edu.au (J.B.); 3 Department of Endocrinology, College of Medicine and Public Health, Flinders University, Adelaide 5042, Australia

**Keywords:** Japanese encephalitis, vaccine, singles dose protection, flaviviruses, antibody-dependent enhancement, Advax, interferon gamma, cell culture-grown inactivated JE vaccine

## Abstract

ccJE+Advax is an inactivated cell culture Japanese encephalitis (JE) vaccine formulated with Advax, a novel polysaccharide adjuvant based on delta inulin. This vaccine has previously shown promise in murine and equine studies and the current study sought to better understand its mechanism of action and assess the feasibility of single dose vaccine protection. Mice immunised with ccJE+Advax had higher serum neutralisation titres than those immunised with ccJE alone or with alum adjuvant. ccJE+Advax induced extraordinarily broad cross-neutralising antibodies against multiple flaviviruses including West Nile virus (WNV), Murray Valley encephalitis virus (MVEV), St Louis encephalitis virus (SLEV) and Dengue virus-1 and -2 (DENV-1 and -2). Notably, the DENV-2 cross-neutralising antibodies from ccJE+Advax immunised mice uniquely had no DENV-2 antibody-dependent infection enhancement (ADIE) activity, in contrast to high ADIE activity seen with DENV-1 cross-reactive antibodies induced by mbJE or ccJE alone or with alum adjuvant. JEV-stimulated splenocytes from ccJE+Advax immunised mice showed increased IL-17 and IFN-γ production, consistent with a mixed Th1 and Th17 response, whereas ccJE-alum was associated with production of mainly Th2 cytokines. In a mouse lethal challenge study against highly virulent JaTH160 JEV strain, ccJE+Advax conferred complete protection in a two-dose schedule with 50 ng of vaccine antigen and near complete protection after a single 200 ng dose of vaccine antigen. There is an ongoing lack of human vaccines against particular flaviviruses, including WNV, SLEV and MVEV. Given its ability to provide single-dose JEV protection and induce broadly neutralising antibodies devoid of ADIE activity, ccJE+Advax vaccine could be useful in situations where rapid protection is desirable, e.g., during a local outbreak or for use in travellers or armies requiring rapid deployment to JEV endemic regions.

## 1. Introduction

Japanese encephalitis virus (JEV) is a flavivirus that is one of the leading causes of viral encephalitis in Asia with over 3 billion people living in JEV endemic regions [1]. JEV is transmitted by *Culex* mosquitoes primarily to birds and pigs which act as natural reservoir for the virus, and then secondarily to humans. An estimated 68,000 human cases of Japanese encephalitis (JE) occur annually resulting in about 16,000 deaths [2]. Most cases of JE (75%) occur in children under 14 years of age [2]. The virus causes acute inflammation in the central nervous system, and a significant portion of survivors (>30%) suffer from permanent neurological, behavioural and cognitive sequelae [3]. A recent study found that 81% of cases occur in areas with JEV vaccination programs which could suggest current JEV vaccines provide less than ideal protection [2].

The first commercially available JE vaccine (JE-VAX) was derived from infected mouse brain tissue (mbJE) and required three-doses to achieve protective immunity in 90% of immunised individuals [4]. However the production and rollout of the vaccine was later halted due to serious side effects including systemic allergic reactions and hypersensitivity [5]. A live attenuated JE vaccine (SA14) was developed in China but still required at least two doses for protection and had issues with the consistency of packaging cell lines and potential carry-over of adventitious agents [6]. In addition, JEV can mutate during passage [7,8,9] raising concerns of phenotypic reversion of the live attenuated virus. More recently, cell culture-grown inactivated JE vaccines (ccJE) have been developed. The ccJE vaccine has an excellent safety record [10], but requires two or more doses to induce protective immunity. JEV is predominately found in rural areas [11] where access to healthcare infrastructure is not as readily available and therefore a JEV vaccine requiring multiple doses may reduce vaccine uptake and population coverage.

JEV belongs to a family of antigenically-related flaviviruses including, West Nile virus (WNV), Murray Valley encephalitis virus (MVEV), St Louis encephalitis virus (SLEV), and Dengue virus (DENV) [12]. This can be both advantageous and disadvantageous as immunity can provide cross protection [13,14,15,16], but can also be associated with antibody-mediated disease enhancement (ADE) [15,16,17,18]. This is particularly important for JEV and DENV as the two strains co-circulate in the same regions of Southeast Asia [19]. Dengue disease enhancement is thought to be mediated by low titres of non-neutralising antibodies resulting in increased uptake of virus into permissive cells expressing Fcɣ receptors, such as monocytes [20]. Hence achieving an adequate magnitude and quality of neutralising antibody response to relevant flaviviruses may be key to preventing disease enhancement.

In addition to the antibody response, a strong cellular immune response is critical in mitigating JEV disease pathology. Adoptive transfer of anti-JEV effector T cells was able to clear virus and protect animals from lethal intracerebral JEV challenge [21]. Additionally, a plasmid DNA JEV vaccine conferred significant protection despite absence of detectable antiviral antibodies, suggesting a role of T cells in protection [22]. Furthermore, an analysis of JEV endemic areas of Southern India showed a significant negative correlation between interferon gamma (IFN-γ) levels and the severity of post-encephalitic neurological sequelae in patients, which suggests IFN-γ may reduce clinical pathology of JEV [23]. Altogether, these findings suggest that a Th1-biased cellular response characterised by high IFN-γ production may be important in immune protection.

The level and type of vaccine-induced immune response can be significantly augmented by the choice of adjuvants. The ideal adjuvant for JE vaccines should be (i) well-tolerated, (ii) safe, (iii) induce high serum neutralising antibody titres and memory B cells without increasing the risk of ADE to related flaviviruses, (iv) induce strong Th1 immunity, and (v) enable single-dose protection. Advax adjuvants are based on non-reactogenic inulin polysaccharide particles and have shown promise in animal and human vaccines [24]. In a previous study, our group demonstrated that a two dose schedule of ccJE formulated with Advax adjuvant was able to boost neutralising antibodies and provide 100% protection of mice from challenge with the JEV Nakayama strain [25].

The purpose of this current study was to test whether it was possible to fully protect against JEV with a single vaccine dose as well as to assess cross-neutralisation activity against other flaviviruses, in particular WNV, SLEV and DENV strains, and assess for any risk of ADE. In addition, given the important role of cellular immunity in mitigating disease pathology of JEV, we examined the effects of Advax adjuvant on the ccJE vaccine induced T cell response. Finally, we tested the ability of the Advax-adjuvanted ccJE vaccine to protect against JaTH160 strain, a new and more virulent JEV strain that has higher mortality and more pronounced virus propagation [26].

## 2. Methods

### 2.1. Animals

All studies were performed in accordance with the guidelines specified by the Animal Experimentation and Ethics Committee of the Kitasato Institute for Life Sciences, Kitasato University (protocols number: 14002). Four-week-old female C57BL/6 mice were obtained from Japan SLC Inc. (Hamamatsu, Japan) and female IFN-γ knockout (KO) mice (B6.129S7-*Ifng^tmlTs^*) were obtained from Oriental Bioservice Inc. (Kyoto, Japan). Animals were maintained under specific pathogen-free conditions.

### 2.2. Cell and Viruses

Vero cell (JCRB9013) was obtained from the Japanese Collection of Research Bioresources Cell Bank (National Institutes of Biomedical Innovation, Health and Nutrition, Osaka, Japan). Working stocks of JEV (Beijing-1, JaTH-160 strain), MVEV (MVE-1-51 strain), DENV-1 (Philippine strain) and -2 (India strain) were 10% suckling mouse brain homogenates in Hanks’ balanced salt solution containing 20 mM HEPES buffer (pH 8.0) and 0.2% bovine serum albumin (HBSS-BSA). WNV (NY-101 strain) and SLEV were grown Vero cells in MEM containing 2% festal bovine serum. Virus titres were determined by plaque formation on Vero cells, as described [27,28].

### 2.3. Immunisation Schedule

Formalin inactivated mouse brain-derived JE vaccine (mbJE: Nakayama strain) and Vero cell culture-grown inactivated JE vaccine (ccJE; Beijing-1 strain) were manufactured by the Kitasato Institute Research Center for Biologicals (Saitama, Japan). Advax delta inulin adjuvant was obtained from Vaxine Pty Ltd. (Adelaide, SA, Australia). C57BL/6 or IFN-γ KO mice (*n* = 10 or 5/group) were immunised twice, 3 weeks apart, with ccJE (50 ng) formulation with Advax (1 mg) or aluminium hydroxide (alum) (30 μg). An additional experimental group of mice were immunised with mbJE (50 ng) for comparison. Blood and spleens were collected 3 weeks after last immunisation for further analysis.

### 2.4. Antibody Isotypes

Isotypes of JEV, WNV and DENV-2-specific antibodies in blood serum samples were determined by the ELISA-based Mouse Typer Sub-Isotyping kit (Bio-Rad, Hercules, CA, USA) according to the supplier’s instructions. JEV, WNV or DENV-2-coated ELISA trays were used and serum samples from immunised mice were diluted 100-fold in Blotto/Tween (Novatein Biosciences, Woburn, MA, USA) and assayed in duplicate. The optical density was measured at 450 nm (OD450). Three naïve control sera were included in each test. To determine relative isotype titres, the mean OD values of test sera were divided by 2 times the corresponding mean OD value of the control sera.

### 2.5. Plaque Reduction Neutralisation Tests

Plaque-reduction neutralisation tests (PRNT_50_) were performed by incubating 200 PFU of JEV (Beijin-1 strain), MVEV (MVE-1-51 stain) or WNV (NY-101 strain) in 110 µL HBSS-BSA with serial 2-fold dilutions of antiserum in the same buffer in a 96-well tray at 37 °C for 1 h. The complement was inactivated by heating the sera at 56 °C for 0.5 h before use. Duplicate 0.1 mL aliquots were assayed for infective virus by plaque formation on Vero cell monolayers grown in 6-well tissue culture trays. The percentage plaque reduction was calculated relative to virus controls incubated with naïve serum from the same mouse strain. Controls yielded 50–100 PFU/well. PRNT_50_ titres were given as the reciprocal of serum dilutions, which resulted in ≥50% reduction of the number of plaques.

### 2.6. Antibody-Dependent Infection Enhancement Assay

DENV-2 ADIE activities were determined by conventional plaque reduction neutralization test against DENV-2 using BHK-FcγRIIA cells provided by the National Institute of Infectious Disease. Fold enhancement values (FEV) and positive infection enhancement were calculated using the following formulas as previously described [29]:

FEV = Mean Plaque with sera (On BHK-FcγRIIA cells)

Mean Plaque w/o sera

Cut-off value = Sum of the mean of negative control wells

Positive Infection Enhancement = FEV > (Cut-off value + 2 standard deviation)

### 2.7. Multiplex Immunoassay for Quantification of Secreted Cytokines

C57BL/6 mice (*n* = 10/group) were vaccinated as per immunisation schedule and spleens were collected 3 week post last immunisation. Splenocytes were restimulated with ccJE vaccine (1 μg/106 cells) or flaviviruses (JEV, WNV, DENV-1 or DENV-2) at a MOI of 0.1 for 10^6^ cells for 4 days. Levels of secreted cytokines in culture supernatant was measured using a Bio-Plex Pro Mouse Cytokine 23-Plex Immunoassay (Bio-Rad) and VeriKine Mouse Interferon Alpha ELISA Kit (PBL Assay Science, Piscataway, NJ, USA) according to manufacturers’ instructions.

### 2.8. Enzyme-Linked Immunospot (ELISPOT) Assay

C57BL/6 mice (*n* = 10/group) were vaccinated as per immunisation schedule and spleens were collected 3 week post last immunisation. Splenocytes were restimulated with 50 ng of ccJE or mbJE vaccine, or with JEV, MVEV or WNV (MOI = 0.01) at a concentration of 5 × 10^5^ cells/well in duplicate overnight at 37 °C. Antigen-specific IFN-γ and IL-17A ELISPOT assays were conducted with Mouse IFN-γ/IL-17 Dual-Colour ELISPOT Kit (R&D Systems) and Mouse IL-5 ELISpot Kit (R&D Systems) respectively, according to the manufacturer’s instructions.

### 2.9. JEV Challenge

C57BL/6 mice (*n* = 10/group) were immunised intramuscularly with ccJE alone or with Advax (1 mg) twice, 1 week apart, with a vaccine antigen dose of 50 ng or once with a vaccine antigen dose of 500 ng or 200 ng. One (double doses) or 2 (single dose) weeks after the final immunisation, mice were challenged via intraperitoneal route with a lethal dose of 3 × 10^2^ PFU JaTH160 strain, corresponding to 20 × LD_50_ and were monitored daily for over 3 weeks.

### 2.10. Statistics

Differences in survival ratios in mouse challenge experiments were assessed using log-rank (Mantel-Cox) test and the Wilcoxon signed-rank test was used to assess differences in antibody titres for significance. Samples with titres below the detection limit of the serological assays were given titres of half that of the detection limit for calculations.

## 3. Results

### 3.1. ccJE+Advax Vaccine Induces Broadly Cross-Neutralising Antibody

Groups of mice were vaccinated with two doses of ccJE with or without Advax or alum adjuvant. An additional group was immunised with a comparable dose of inactivated mouse brain JE antigen (mbJE). Serum was obtained 3 weeks after the last immunisation, pooled for each group to provide sufficient serum to run all assays and then assayed for its ability to neutralise JEV and the other flaviviruses (WNV, MVEV, SLEV and DENV serotypes 1 and 2). mbJE induced the highest titres of neutralising antibodies against the homologous JEV but induced low or undetectable cross-neutralising antibodies against the other flaviviruses (Table 1(iv)). Similarly, ccJE alone induced high neutralising antibodies against homologous JEV but low cross-neutralising antibodies against the other flaviviruses (Table 1(iii)). The best overall cross-neutralising responses against all flaviviruses were achieved by immunisation with ccJE+Advax which induced detectable neutralising antibody titres against all the tested flaviviruses, namely, JEV, WNV, MVEV, SLEV, DENV-1 and DENV-2 (Table 1(i)). A neutralisation titre of 1:10 is considered seroprotective for flaviviruses [30], indicating that ccJE+Advax was able to induce protective levels of cross-neutralising antibodies against this highly divergent group of flaviviruses, except for SLE St Louis encephalitis virus (SLE), where it induced low but detectable levels of neutralising antibodies. Indeed, for most of flaviviruses, ccJE+Advax induced neutralisation titters almost 10 times higher than the sero-protection cut-off. The order of ranking of neutralisation from highest to lowest in this vaccine group was JEV > DENV-2 > MVEV > DENV-1 > WNV > SLEV. These results indicate that Advax adjuvant, when formulated with ccJE antigen, is able to induce broadly cross-reactive neutralising antibodies against a wide range of flaviviruses.

### 3.2. ccJE+Advax Stimulates a Balanced Th1/Th2 Antibody Response

**Figure 1:**
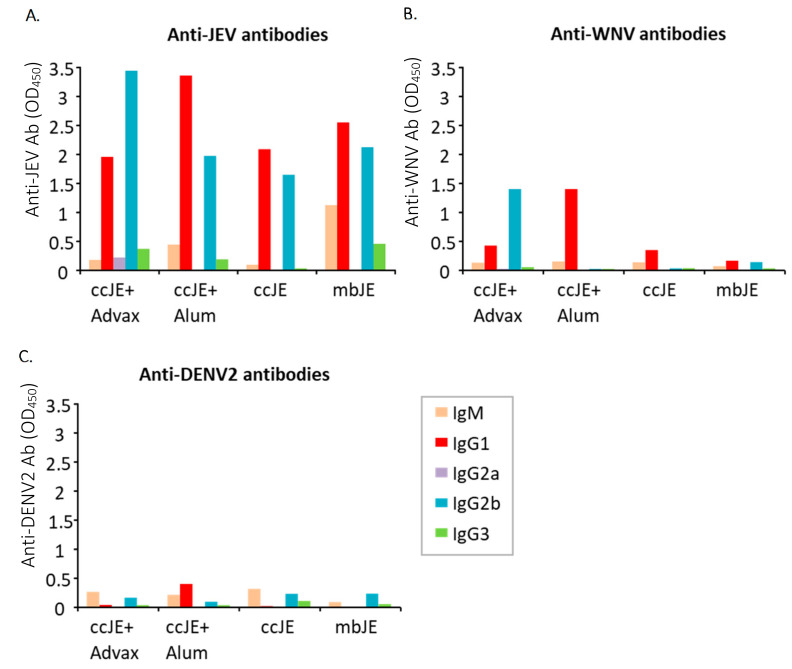
Advax induces a Th1-dominant antibody response. C57BL/6 (*n* = 10/group) were immunised and boosted after 3 weeks with ccJE (50 ng) or mbJE (50 ng) alone or with Advax (1 mg) or alum (30 µg). Blood was collected 3 weeks post last immunisation and antigen-specific antibody titres against (**A**) JEV, (**B**) WNV and (**C**) DENV2 were determined by ELISA using isotype/subclass-specific antibodies on pooled serum for each group and are shown as OD values at 450 nm.

Immunised mouse sera were tested for JEV (Beijing-1 strain) antibody subtype binding by ELISA. ccJE+Advax induced higher production of IgM and IgG subtypes with the exception of IgG1 when compared to immunisation with ccJE alone (Figure 1A). Consistent with the neutralising antibody results, only ccJE+Advax immunised mice showed both WNV-binding IgG1 and IgG2b, with low to undetectable levels of anti-WNV antibodies in sera from animals immunised with ccJE or mbJE alone (Figure 1B). DENV-2 binding activity was very low overall for all groups, with IgM the predominant isotype detected (Figure 1C). Overall, ccJE+Advax elicited a balanced but slightly Th1 skewed antibody subtype response, with a higher ratio of IgG2b to IgG1, whereas ccJE+alum induced a lower IgG2b to IgG1 ratio, consistent with alum inducing a Th2 biased immune response (Table 2(i–iii)).

To further study the potential role of Advax-specific differences in Th1/Th2 immune bias in induction of broadly neutralising antibodies by the different JEV vaccine formulations, we repeated the JEV immunisations in an IFN-γ knockout (KO) mouse model (Table 1(iv,v)). IFN-γ has been shown to increase Th1-type antibody isotype production [31] and in B6.129S7-*Ifng^tmlTs^* mice this IFN-γ-driven Th1 antibody isotype switching has been shown to be abrogated [32]. Analysis of the anti-JEV IgG isotypes again showed no impact of the absence of IFN-γ on the ability of Advax to enhance IgG2b responses to the ccJE antigen, when compared to either ccJE or mbJE alone and did not affect the ability of ccJE+Advax to generate high titres of WNV cross-reactive antibodies. As expected, the Th2 bias of mbJE and ccJE as evidenced by a low IgG2b:IgG1 ratio was maintained in the IFN-γ KO mice. The fact that the high IgG2b:IgG1 ratio was preserved in the JE-Advax immunised IFN-γ KO mice was an interesting finding as it indicates that the ability of Advax to induce an IgG2b (Th1-type) isotype switch is not dependent on the Th1 cytokine, IFN-γ.

### 3.3. Antibody-Dependent Infection Enhancement

Dengue serotype specific ADE has been a major obstacle in the development of dengue vaccines [33] and has also been shown, at least experimentally, for other flaviviruses such as JEV [34]. While initially described as a consequence of previous natural infection, ADE has also been observed after dengue immunisation. As our ccJE+Advax vaccine induced high levels of cross-neutralisation of DENV-1 and -2, we wished to see whether this cross-reactive antibody might be associated with potential antibody-dependent infection enhancement (ADIE) for dengue virus that could potentially lead to ADE. DENV-2 neutralisation and ADIE were compared using a conventional plaque reduction neutralisation assay using BHK cell or BHK-FcγRIIA cell lines, respectively. Immune sera of mice immunised with ccJE-Advax demonstrated high levels of DENV-2 neutralising activity in both the BHK (PRNT 1.37) and BHK-FcγRIIA (PRNT 1.31) cell lines, but with no evidence of ADIE activity (Table 3). By contrast, all immune sera of mice immunised with ccJE or mbJE alone, or ccJE+alum, demonstrated significant levels of ADIE activity. In particular, the mbJE-immune sera which had no DENV-2 neutralising activity, had the highest ADIE activity, causing over 11-fold increased infection of BHK-FcγRIIA cells. The next highest ADIE activity was seen in sera from the ccJE+alum immunised mice. Altogether, the results indicate that Advax adjuvanted ccJE is able to induce broadly cross-reactive neutralizing antibody against a wide range of flaviviruses, but these cross-reactive antibodies, unlike antibodies in mice immunised with vaccines not containing Advax, do not mediate ADIE against DENV2.

### 3.4. Cellular Immune Response

**Figure 2:**
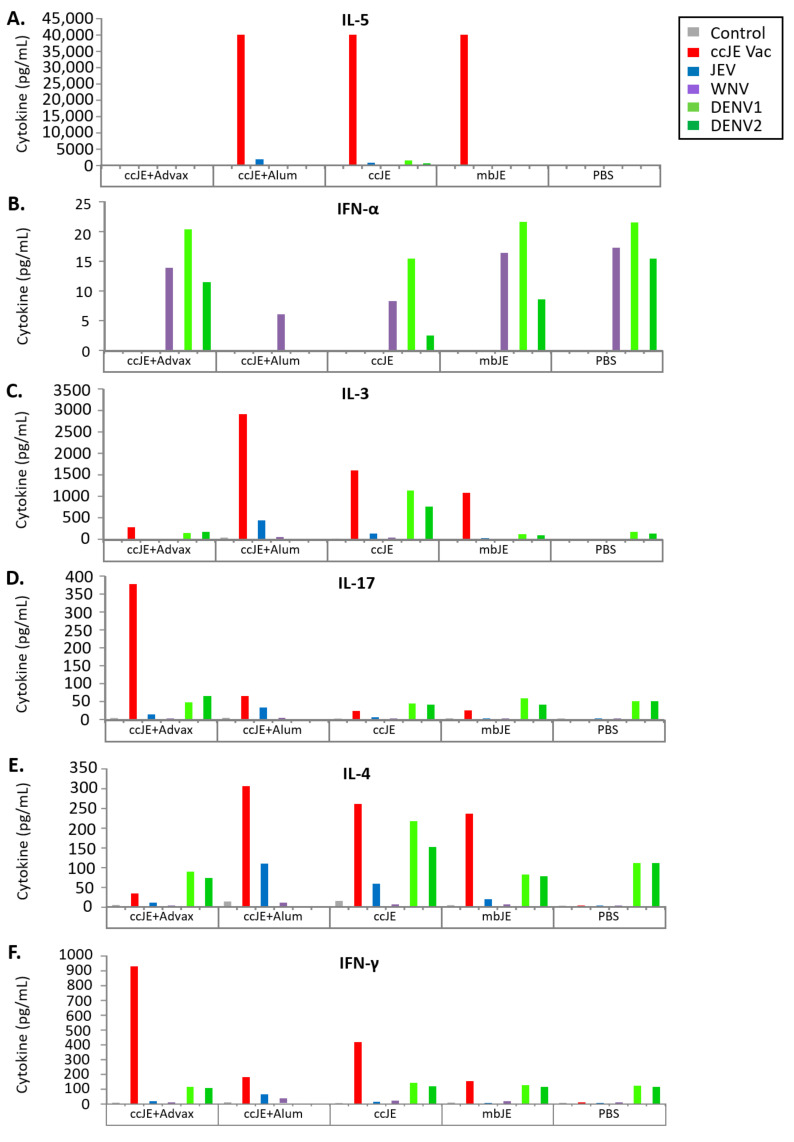
Addition of Advax enhances Th1-associated cytokine production to ccJE vaccine. C57BL/6 mice (*n* = 10/group) were immunised and boosted after 3 weeks with ccJE (50 ng) or mbJE (50 ng) alone or formulated with Advax (1 mg) or alum (30 µg). Spleens were collected 3 week post last immunisation and pooled for each group. Splenocytes were stimulated with ccJE vaccine (1 μg/10^6^ cells) or flaviviruses (JEV, WNV, DENV1 or DENV2) at a MOI of 0.1 for 10^6^ cells for 4 days. Cytokines, (**A**) IL-5, (**B**) IFN-α, (**C**) IL-3, (**D**) IL-17, (**E**) IL-4 and (**F**) IFN-γ were measured in culture supernatant using a mouse multiplex immunoassay and a mouse interferon-α ELISA kit.

Cellular immunity has been shown to be important for protection against JEV and other flaviviruses. Therefore, the amount of secreted cytokines in pooled splenocytes from each group in recall response to stimulation with either inactivated or live homologous or heterologous flavivirus antigens was assessed by mouse cytokine multiplex immunoassays (Bio-Plex) and mouse IFN-α ELISA kit (Figure 2). Immunisation with ccJE+Advax markedly increased IL-17 and IFN-γ response to ccJE but not to the other flaviviruses (Figure 2D,F). Immunisation with ccJE, and to a lesser extent mbJE, increased production of the Th2 cytokines, IL-3, IL-4 and IL-5 after virus stimulation (Figure 2A,C,E). By contrast, no increase in the IL-3, IL-4 or IL-5 response to any of the flaviviruses was seen in the ccJE+Advax group, consistent with ccJE+Advax biasing towards a Th1 response. The IFN-α response to all flaviviruses was suppressed in the ccJE+alum and to a lesser extent, ccJE alone group (Figure 2B). In addition, an ELISPOT assay to assess the frequency of cytokine-producing cells following re-stimulation with either inactivated or live homologous or heterologous flavivirus antigens showed Advax increased the frequency of IFN-γ producing cells against a broad range of antigen stimuluses when compared to other groups, with the only exception of ccJE+alum in response to mbJE antigen (Supplementary Figure S1A). IL-17 producing cells were highest in ccJE+Advax immunised mice, whilst the number of IL-5 producing cells was highest in ccJE+alum or mbJE alone immunised mice (Supplementary Figure S1B,C). This shows that the choice of adjuvant is important in shaping the cytokine profile of antigen-specific immune cells. Given the important role of cytokines, such as IFN-γ in flavivirus protection [35], this suggests Advax may be ideally suited for use as adjuvants in flavivirus vaccines.

### 3.5. ccJE-Advax Provides Robust JEV Protection

**Figure 3:**
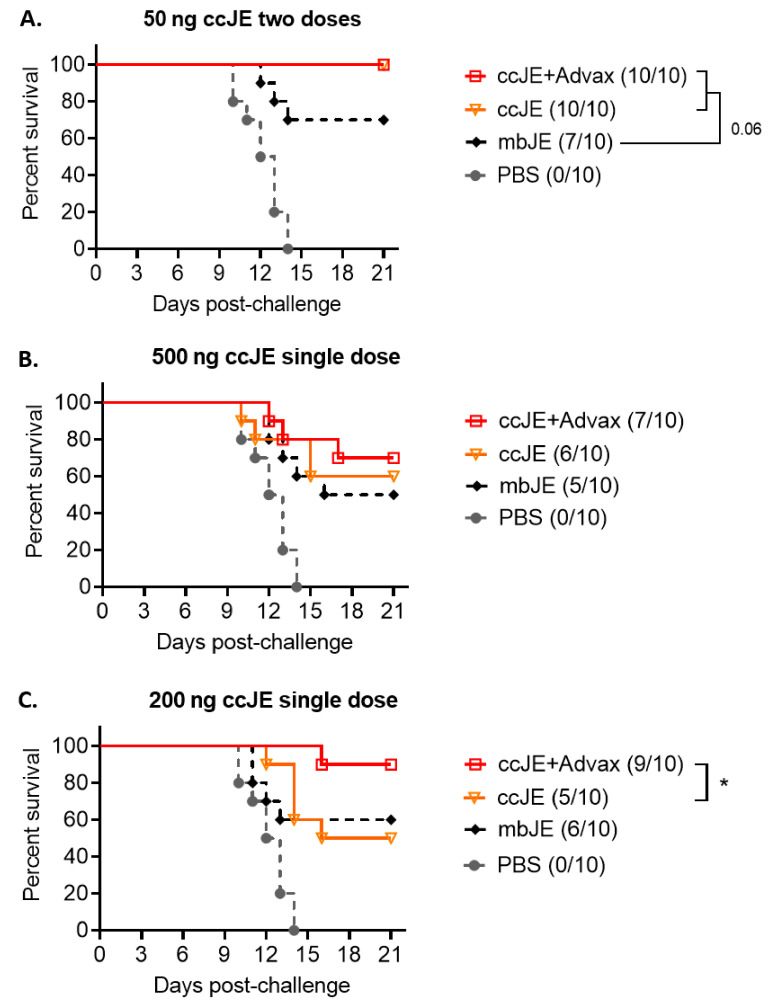
Advax adjuvanted ccJE vaccine provides robust protection against JEV. Four-week-old C57BL/6 mice (*n* = 10/group) were immunised intramuscularly with ccJE alone or with Advax (1 mg) (**A**) twice, 1 week apart, with a vaccine antigen dose of 50 ng or once with a vaccine antigen dose of (**B**) 500 ng or (**C**) 200 ng. As a control, mice were also immunised mbJE or PBS. One week after the last immunisation, mice were challenged intraperitoneally with 3 × 10^2^ PFU JEV JaTH160 strain. Survival rate (number of survivors/number of challenged mice shown in parenthesis, * *p* < 0.05 by log-rank Mantel-Cox test).

Given the Advax ability to induce a strong cellular response marked by enhanced IFN-γ production and broad cross-neutralising antibodies against other flaviviruses without enhancement of DENV-2 ADIE, we chose to focus on the Advax adjuvant for further vaccine development. To assess whether ccJE+Advax can confer protection against JaTH160, a new and more virulent JEV strain, mice were immunised intramuscularly twice, 1 week apart, with either inactivated mbJE or ccJE alone or formulated with Advax. One week after the final immunisation, mice were challenged via intraperitoneal route with 3 × 10^2^ PFU JaTH160 strain, equivalent to 20 × LD_50_. Furthermore, 100% of control PBS-immunised mice succumbed to infection within 2 weeks. Mice that received two doses of ccJE antigen alone or combined with Advax adjuvant formulations were 100% protected, whereas only partial survival (70%) was observed in the mice that received two doses of mbJE alone (Figure 3A).

Next, we tested whether inclusion of Advax adjuvant formulations with ccJE could provide single-dose vaccine protection. Mice at 4 weeks of age were immunised with a single dose of 500 ng or 200 ng mbJE or ccJE antigen formulated with Advax, and challenged 2 weeks later with 3 × 10^2^ PFU JaTH160 strain. After the 500 ng single dose, 70% protection was seen in the groups immunised with Advax with slightly lower protection for ccJE (60%) or mbJE (50%), alone (Figure 3B). Interestingly for the single 200 ng dose study, the survival in the ccJE+Advax group increased to almost complete level of protection (90%), whilst mbJE (60%) and ccJE (50%) alone largely remained unchanged (Figure 3C). This shows that Advax adjuvant is capable of conferring robust JEV vaccine protection, even after just a single dose, and that the ratio of vaccine antigen and adjuvant is important.

To assess for a correlation between neutralising antibody titres and protection, sera was collected prior to challenge from mice that received either single (200 ng and 500 ng) or two dose (50 ng) vaccination regimens. Neutralising titres against the Beijing JE strain were highest in mice that received two doses of vaccine. This was followed by the 500 ng single dose groups with lowest responses in the 200 ng single dose groups (Table 4). There was a correlation between neutralising titre and protection in the twice immunised groups with the mbJE group having the lowest titres and also lowest survival (60%). However, this relationship did not hold true in the 200 ng single dose group where the ccJE+Advax group had a relatively modest PRNT of 0.97, just under the accepted seroprotection level of 1, but had the highest survival (90%). Moreover, the mbJE group had the highest PRNT of 1.43 in the 200 ng vaccine antigen single dose schedule, but achieved only 60% survival. Hence, the serum neutralising antibody levels in the single immunisation groups showed a poor correlation with the challenge outcome. This suggests that Advax may enhance single dose protection by alternative mechanisms such as via increased numbers of memory B cells, change in functional antibodies or enhanced cellular immunity.

## 4. Discussion

Advax is a novel polysaccharide adjuvant based on microparticles of delta inulin, which potently stimulates vaccine immunogenicity whilst being safe and non-reactogenic [24]. Advax is distinct from typical vaccine adjuvants as it does not appear to work through induction of inflammatory danger signals, but rather potentiates the intrinsic or in-built adjuvant property of co-administered antigens [36]. Vaccines containing Advax adjuvant have been extensively evaluated in human clinical trials, including in hepatitis B [37], influenza [38,39,40], insect-sting allergy [41] and SARS-CoV-2 vaccines. Advax adjuvant has previously been shown to enhance ccJE vaccine immunogenicity in mice [42] and horses [25]. The current study explored the mechanisms behind how Advax enhances ccJE vaccine responses including assessing the breadth of cross-reactivity against other flavivirus family members, potential for such antibodies to induce ADIE and tested whether Advax adjuvant would allow single-dose vaccine protection against a high virulence JEV strain.

In our study, ccJE formulated with traditional alum adjuvant induced predominately IgG1 antibody which conforms with existing literature that alum adjuvant imparts a major Th2 bias in vaccine responses [43]. A strong Th2 bias was also seen after immunisation with ccJE or mbJE alone. On the other hand, ccJE formulated with Advax produced a balanced Th1 and Th2 response as demonstrated by induction of approximately equal amounts of both IgG1 and Ig2b. Splenocytes isolated from mice immunised with mbJE or ccJE alone or ccJE+alum and re-stimulated in vitro produced the highest amounts of Th2 cytokines (IL-3, IL-4, IL-5), whereas immunisation with ccJE+Advax resulted in increased production of both Th1 and Th17 cytokines (IFN-γ and IL-17). This is consistent with Advax adjuvant imparting a significant Th1 bias to the immune response to the ccJE antigen.

An interesting feature not previously reported is the different pattern of antibody responses induced by our new ccJE vaccine formulation when compared to the traditional mbJE vaccine. While the mbJE antigen induced high levels of neutralising antibodies to the homologous JE virus, it failed to induce cross-neutralising antibodies against the other flaviviruses. By contrast, ccJE+Advax induced high levels of cross-neutralising antibodies against a broad range of flaviviruses including WNV, MVEV, DENV-1, DENV-2, and even low neutralisation of SLEV. Based on a review of current literature, no other flavivirus vaccine approach seems to have demonstrated such extensive flavivirus cross-protection using a single flavivirus antigen. Inactivated JE vaccines, JE-VAX [44] or live SA14-14-2 JE vaccine [45], were previously reported to induce undetectable or non-protective levels of neutralising antibody against MVEV and WNV, consistent with our findings for mbJE vaccine. This difference suggests that our ccJE antigen presents to the immune system one or more unique flavivirus-neutralising epitopes not presented by the mbJE antigen, and recognition of these additional epitopes is enhanced by formulation with Advax adjuvant.

Notably, the induction of DENV binding and neutralising antibodies by ccJE+Advax did not induce infection enhancement for DENV-2 in the in vitro ADIE cellular assays. DENV ADE is a high risk in the presence of sub-neutralising antibodies induced by other DENV sub-strains or related flaviviruses. Only ccJE+Advax showed no signs of DENV-2-induced ADIE in the BHK-RcγRIIA cell assay. Notably, immune sera from all other immunisation groups including ccJE alone, ccJE-alum and mbJE demonstrated lower neutralisation and increased ADIE activity in the same BHK-RcγRIIA assay. This indicates that not only is Advax adjuvant able to induce high levels of flavivirus cross-neutralisation, but that the antibodies that mediate this function uniquely do not cause enhanced infectivity, either because of their potent neutralisation capacity or because they are in some other way functionally different to antibodies induced by alum adjuvant or the antigens by themselves.

The recall response studies showed that Advax developed a Th1-, and to lesser extend Th17-, dominant cellular phenotype. Other long-term studies of Advax adjuvants have shown that this T cell IFN-γ recall response continues to become progressively stronger up to a year post-immunization (unpublished data). We currently do not know whether this is due to progressive conversion over time of other memory T-cell subsets, e.g., Th0, Th2 and Th17 cells into IFN-γ producing Th1 cells, continued proliferation and expansion of an IFN-γ producing memory Th1 cell subset, or ongoing activation by long-term antigen depots of naïve T cells into IFN-γ producing Th1 cells.

Due to extensive Th2 bias with alum adjuvant and potential risk of DENV ADIE leading to ADE, we focused on evaluating ccJE with Advax against highly virulent JaTH160 JEV strain. In our study, the two-dose regime at 50 ng was able to confer complete protection against JaTH160. The addition of the Advax adjuvant enabled near complete protection with a single 200 ng dose of ccJE antigen. To the best of our knowledge, we are the first group to boost the immunogenicity of ccJE through use of an adjuvant (Advax), thereby enabling robust single-dose protection against lethal JEV infection. By comparison, a two-dose vaccination schedule in human subjects with the mouse brain derived JE-VAX vaccine failed to generate detectable JE neutralising antibody in ~20% of vaccine recipients [46] and eventually three doses were required to achieve adequate immunity [47]. Similarly, inactivated ccJE vaccines (unadjuvanted) are approved in Japan as three dose paediatric schedules [48] with subsequent periodic boosting presumed to occur due to endemic JE exposure. Moreover, an alum-adjuvated ccJE traveller vaccine (called Jespect or Ixaro) has been licensed but also requires a two-dose vaccination schedule [49].

In the single dose group, analysis of sera showed that there was a poor correlation between neutralising antibody levels and protection, in particular, in the single 200 ng dose vaccine antigen schedule, the Advax adjuvanted group had the lowest neutralisation titre but near complete protection. Future studies are required to determine the exact mechanism by which the addition of Advax improved JEV protection. Notably, if these results successfully translate to human subjects, a single dose vaccine based on ccJE+Advax, in addition to significantly reducing vaccine production and rollout costs, could be useful to induce initial JEV immunity in endemic regions as well as for use as a traveller vaccine.

A limitation of the study was that the vaccine formulations were only evaluated in a single preclinical model, namely, inbred mice. Despite species-specific differences with humans, mice are one of the most extensively utilised and characterised animal models for JEV and other flaviviruses, due to their high degree of susceptibility to flavivirus encephalitis and the similarity in disease presentation and virus tropism between rodents and humans [50,51]. This enables us to directly benchmark our results with the findings of other research groups in the literature. In addition, a previous study [25] published by our lab evaluated ccJE with Advax in horses where the cross-neutralisation antibody activity correlated well with observed immunogenicity in our parallel mouse studies. This provides confidence that the single-dose vaccine protection strategy in the current study may be applicable in large animal models and humans.

## Figures and Tables

**Table 1:** Advax induces broad cross-neutralising antibodies against Japanese encephalitis virus (JEV) and other flaviviruses.

	Challenge Virus					
Immunised Mouse Sera	JEV	WNV	MVEV	SLEV	DENV1	DENV2
(i) ccJE+Advax	2.67	1.20	1.87	0.37	1.21	1.91
(ii) ccJE+alum	2.99	0.96	1.45	N.D.	0.89	1.81
(iii) ccJE	2.89	0.87	1.45	N.D.	1.02	1.56
(iv) mbJE	3.37	N.D.	1.19	N.D.	N.D.	0.74

C57BL/6 mice (*n* = 10/group) were immunised and boosted after 3 weeks with mbJE alone and ccJE alone or with Advax or alum. Blood was collected 3 week post last immunisation to assess neutralisation activity. To provide sufficient sera for all assays, all sera for each group was pooled into a single sample. Challenge viruses included JEV, West Nile virus (WNV), Murray Valley encephalitis virus (MVEV), St Louis encephalitis virus (SLEV), and Dengue virus 1 and 2 (DENV1/2). Neutralisation titres are presented as log_10_. Data shown represents pooled sera samples. N.D.: Not detected.

**Table 2:** Advax induces a high IgG2b/IgG1 antibody ratio that is maintained in IFN-γ KO mice. C57BL/6 (*n* = 10/group) and IFN-γ KO (*n* = 5/group) mice were immunised and boosted after 3 weeks with ccJE (50 ng) or mbJE (50 ng) alone or with Advax (1 mg) or alum (30 µg). Blood was collected 3 weeks post last immunisation and antigen-specific antibody titres against JEV, WNV and DENV2 were determined by ELISA using isotype/subclass-specific antibodies on pooled serum for each group and are shown as IgG2b/IgG1 ratios.

Host Mouse	Antigen Specific Antibodies	Immunised Mouse Sera (IgG2b/IgG1 Ratio)			
		ccJE+Advax	ccJE+Alum	ccJE	mbJE
(i) Wild Type	JEV	1.75	0.58	0.79	0.82
(ii) Wild Type	WNV	3.28	0.02	0.10	0.85
(iii) Wild Type	DENV2	3.6	0.24	9.2	11.5
(iv) IFN-γ KO	JEV	1.87	0.36	0.53	0.22
(v) IFN-γ KO	WNV	2.46	0.14	0.26	0.10

**Table 3:** ccJE with Advax induced neutralising antibody against DENV2 without triggering Fcγ receptor antibody-dependent infection enhancement (ADIE) activity.

Immunised Mouse Sera	DENV2			
	(A) Plaque-Reduction Neutralisation Test (PRNT_50_)		(B) Infection Enhancement	
	BHK	BHK-FcγRIIA	BHK	BHK-FcγRIIA
(i) ccJE+Advax	1.37	1.31	0.07	0.15
(ii) ccJE+alum	1.13	N.D.	0.33	3.06
(iii) ccJE	1.51	N.D.	0.20	2.21
(iv) mbJE	N.D.	N.D.	0.66	11.31

C57BL/6 (*n* = 10/group) mice were immunised and boosted after 3 weeks with ccJE (50 ng) or mbJE (50 ng) alone or with Advax (1 mg) or alum (30 µg). Blood was collected 3 weeks post last immunisation. (A) Plaque-reduction neutralisation tests (>50% method) (PRNT_50_) with immunised mouse sera using BHK cell or BHK-FcγRIIA cells against DEVN2. PRINT_50_ titres are presented as log_10_. (B) Fold enhancement values. immunised mouse sera using BHK cell or BHK-FcγRIIA cells and DENV2. Underline indicates infection-enhancement activity (see Methods section for calculating fold-enhancement, cut-off value and infection enhancement activity). N.D.: Not detected.

**Table 4:** Advax adjuvants induce strong neutralising antibodies against JEV in either a single or two dose vaccine regimen.

Immunised Mouse Sera	JEV		
	Single		Double
	500 ng	200 ng	50 ng
(i) ccJE+Advax	1.972	0.967	2.512
(ii) ccJE	1.182	0.786	2.098
(iii) mbJE	0.966	1.433	1.343

Four-week-old C57BL/6 mice (*n* = 10/group) were immunised intramuscularly with ccJE 50 ng or with Advax (1 mg) twice, 1 week apart, or once with ccJE 500 ng or 200 ng with the same adjuvants. Blood was collected at week 2. Data shown represent pooled sera samples for each group. Neutralisation titres are presented as log_10_.

## Data Availability

The data presented in this study are available on request from the corresponding author.

## Supplementary Materials

The following are available online at www.mdpi.com/article/10.3390/vaccines9111235/s1, Figure S1: Advax induces strong IFN-γ cellular response. Four-week-old C57BL/6 mice were immunised intramuscularly with mbJE or ccJE alone or with Advax or alum twice 3 weeks apart with a vaccine antigen dose of 50ng. Spleens were collected 3 weeks after the last immunisation. Antigen-specific (A) IFN-γ, (B) IL-17 and (C) IL-5 producing splenocytes were evaluated by ELISPOT following restimulation with 50ng of ccJE or mbJE vaccine, or with JEV, MVE or WNV (MOI = 0.01) for 24 h.
