# Supplementary material for: An Advax-Adjuvanted Inactivated Cell-Culture Derived Japanese Encephalitis Vaccine Induces Broadly Neutralising Anti-Flavivirus Antibodies, Robust Cellular Immunity and Provides Single Dose Protection
Source: Vaccines (Basel). 2021 Oct 23;9(11):1235. doi: 10.3390/vaccines9111235 (PMC8618450; doi:10.3390/vaccines9111235)
Supplement: Supplementary file 1 [file vaccines-09-01235-s001.zip › vaccines-1324409-supplementary.pdf]

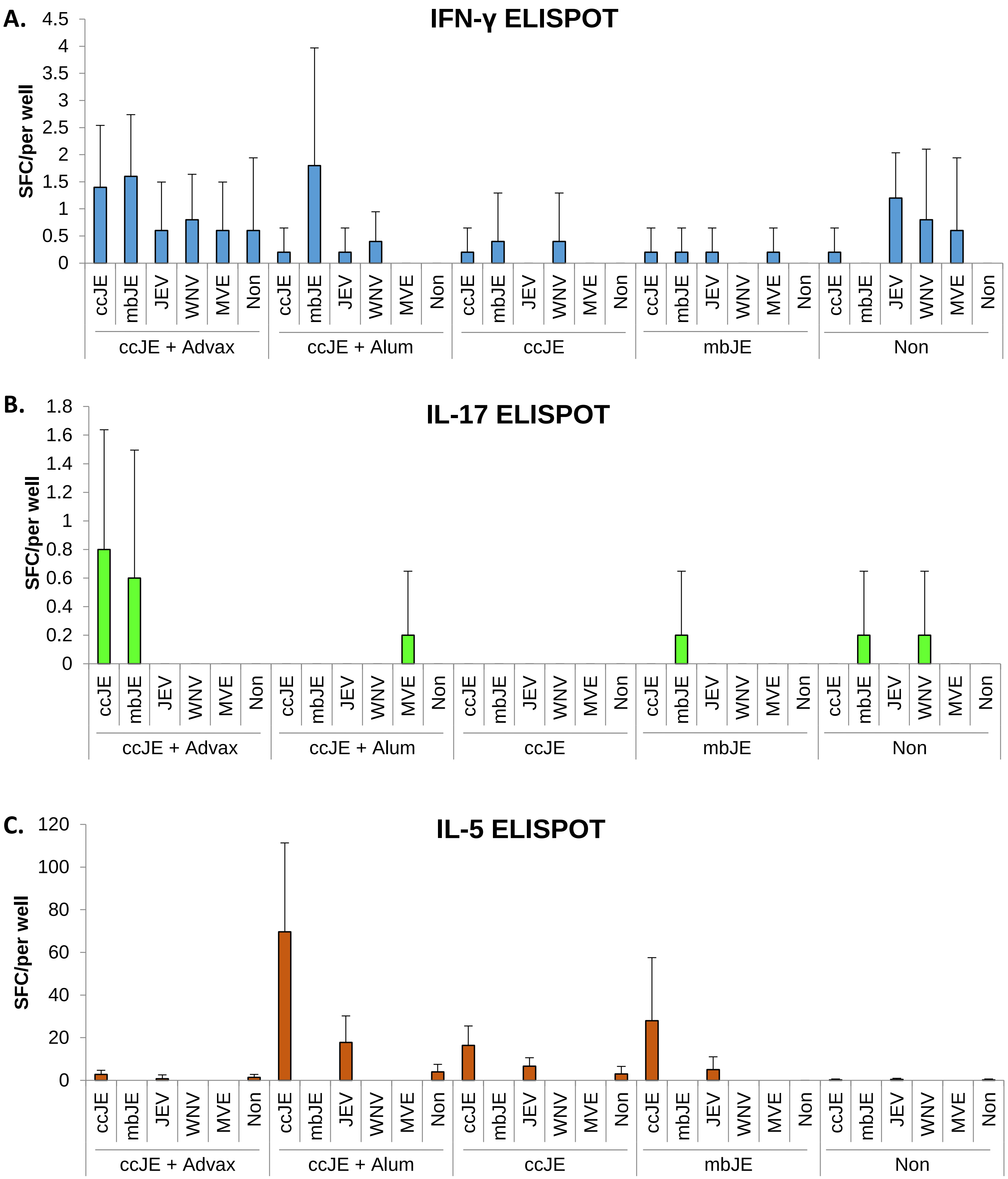

Supplementary Figure S1: Advax induces strong IFN-γ cellular response. Four-week-old C57BL/6 mice were immunised intramuscularly with mbJE or ccJE alone or Advax or Alum twice 3 weeks apart with a vaccine antigen dose of 50ng. Spleens were collected 3 weeks after the last immunisation. Antigen-specific **(A)** IFN-γ, **(B)** IL-17 and **(C)** IL-5 producing splenocytes evaluated by ELISPOT following restimulation with 50ng of ccJE or mbJE vaccine, or with JEV, MVE or WNV (MOI=0.01) for 24hrs.
